# Liquor for breakfast, fighting against alcohol consumption

**DOI:** 10.1192/j.eurpsy.2024.842

**Published:** 2024-08-27

**Authors:** N. Navarro Barriga, P. Marqués Cabezas, M. B. Arribas Simón, B. Rodríguez Rodríguez, C. Alario Ruiz, G. Guerra Valera, A. Aparicio Parras, M. J. Mateos Sexmero, M. Fernández Lozano, P. Martínez Gimeno, M. A. Andreo Vidal, M. Calvo Valcárcel, M. P. Pando Fernández, M. D. L. A. Guillén Soto, T. Jimenez Aparicio, M. D. C. Vallecillo Adame, C. De Andrés Lobo

**Affiliations:** Hospital clínico universitario de Valladolid, Valladolid, Spain

## Abstract

**Introduction:**

The harmful consumption of alcohol is known for how tortuous its management can be in mental health, encouraging introspection of it as a serious problem is perhaps the main key to starting to battle against its damaging influence on the development of a functional and full life.

**Objectives:**

To describe a clinical case showing an unpredictible complication in an alcohol detoxification process.

**Methods:**

54-year-old man, native of Cádiz, widowed for half a decade, without children. He resides with his parents in the family home. Currently unemployed for approximately a year. He has previously worked in the IT sector. As a notable somatic history, we found long-established arterial hypertension and a total hip replacement. He has been under irregular follow-up with a mental health team for anxiety-depressive symptoms in the context of grief. He goes to the emergency service brought by his family to begin the detoxification process in the hospital setting. He acknowledges ethanol consumption since he was widowed, which began when he awakes; quantities that ranged between one or up to three bottles of distilled liquor per day, generally consumption is in the home environment. A little less than a year ago, he began to isolate himself in his room and abandon his self-care, eating increasingly insufficient food intake, refusing to receive professional care to quit the habit, mainly because he did not recognize it as disruptive.

The patient was admitted to hospital with symptoms suggestive of withdrawal, making it extremely difficult to control blood pressure levels. On the third day of admission to the acute care unit, fever peaks, blood pressure levels well below normal parameters, and compromised level of consciousness began to be evident.

**Results:**

Blood tests were performed that, together with the clinical picture, suggested imminent septic shock, so critical care was contacted for transfer and stabilization. A germ of probable urinary etiology sensitive to a broad spectrum of antibiotics was isolated in blood cultures, and the medication of the detoxification process was progressively optimized. Once clinical stability was achieved at all levels, an inpatient cessation resource was managed, which the patient accepted and considered suitable for his complete recovery.

**Conclusions:**

A holistic approach to the alcoholic patient is important, since serious problems of an organic nature often arise. This is why a multidisciplinary intervention is necessary, as well as a holistic approach to care, involving both classic pharmacology and assiduous long-term psychotherapeutic intervention.

**Disclosure of Interest:**

None Declared

